# Examining the role of physical activity in older adults with epilepsy^☆^

**DOI:** 10.1016/j.ebr.2025.100756

**Published:** 2025-02-17

**Authors:** Stephen P. Klaus, Serdar Akkol, Smitha K. Achuthan, Annie He, Cynthia Zheng, Ed Faught, Halley B. Alexander

**Affiliations:** a Trinity College Dublin, College Green, Dublin, Ireland; b University of Alabama at Birmingham, 1670 University Boulevard, Birmingham, AL 35233, USA; c UT Southwestern Medical Center, 5323 Harry Hines Blvd, Dallas, TX 75390, USA; d University of Minnesota, 420 Delaware St SE, Minneapolis, MN 55455, USA; e Emory University, 1365 Clifton Rd, Atlanta, GA 30322, USA; f Wake Forest University School of Medicine Medical Center Boulevard Winston-Salem, NC 27157, USA

**Keywords:** Epilepsy, Older adults, Physical activity, Non-pharmacological treatments, Seizure control, Practice guidelines, Fall prevention, Comorbidities, Multidisciplinary care, Quality of life, Aging

## Abstract

Epilepsy disproportionately affects older adults due to acquired conditions including stroke, neurodegeneration and head trauma secondary to falls. Current literature lacks adequate representation of specific therapies and considerations for this cohort. Furthermore, older adults are more susceptible to the adverse effects of anti-seizure medications necessitating increased caution when treating. Non-pharmacological interventions, including physical activity (PA), are underrecognized, particularly in older adults where they may be of greatest benefit. The following narrative review describes how older adults are uniquely impacted by epilepsy and associated comorbidities. It examines the current literature with respect to PA in epilepsy and, where available, evidence for PA in older adults. This includes how PA can affect pathogenesis and reduce the incidence of epilepsy onset through the reduction of neuroinflammation. PA may also be utilized by older adults with epilepsy to improve cardiovascular function, seizure control, prevent falls and secondary head injury, as an adjunct treatment for mood disorders and cognitive decline, and to promote general well-being. PA has a large and underappreciated role to play in older adults with epilepsy and is increasingly being recognized by healthcare providers and incorporated into practice guidelines.

## Highlights

• Older adults with epilepsy require unique considerations in their treatment.
• Evidence for physical activity (PA) in older adults with epilepsy remains limited.
• PA likely has unique benefits for older adults with epilepsy.
• PA may reduce the incidence of epilepsy in older adults.
• Existing evidence shows PA prevents falls and reduces neuroinflammation.

## 1 Introduction

The incidence of epilepsy rises after the age of 60, with a significantly higher prevalence among older adults compared to younger individuals. Major contributing factors include cerebrovascular disease and neurodegeneration, both of which are more common in older adults [1].

Given the rapidly aging population, it is increasingly important to address the distinct needs of older adults with epilepsy. People with epilepsy (PWE) in this age group often exhibit unique responses to epilepsy treatment and require special consideration regarding drug choice, dosing, and side effects [2]. Importantly, several common causes of epilepsy in older adults can be mitigated, suggesting that the incidence of epilepsy in this population could be reduced through targeted primary prevention efforts. Furthermore, seizures in older adults exacerbate existing health challenges by increasing the risk of fall-related injuries, and worsening comorbid conditions such as cognitive impairment, mood disorders, and cardiovascular disease. Therefore, strategies to reduce the overall incidence of epilepsy and frequency of seizures in this demographic warrant further exploration. Specifically, physical activity (PA) stands out as a promising but underutilized preventive and therapeutic approach for older adults with epilepsy.

Despite the many benefits of PA, PWE remain more sedentary and less physically active than the general population [3]. The Department of Health and Human Services current *Physical Activity Guidelines for Americans* recommend 150 min of moderate-intensity aerobic physical activity a week, or 75 min of vigorous-intensity aerobic physical activity per week, as well as muscle strengthening activities on two or more days per week [4]. Moreover, most older adults (65 years and older) do not meet these recommended levels of PA, resulting in negative effects on health, as well as worsening disability and quality of life (QOL) [5], [6].

Anti-seizure medications (ASMs) are first-line treatment for the management of seizures but are often accompanied by side effects that can worsen existing epilepsy-associated comorbidities such as cognitive impairment, mood disorders, sleep disturbance, and cardiovascular disease. ASM side effects are generally more pronounced in older adults due to physiological changes of aging including reduced hepatic and renal clearance and delayed or decreased absorption, as well as underlying comorbid conditions and polypharmacy [2]. PA, on the other hand, has been shown in other populations to improve mood, cardiovascular health, sleep, cognition and QOL. In the epilepsy population, several small studies have also shown the positive effects of PA on these outcomes for PWE. Therefore, this area is ripe for further research as PA in older adults may help improve seizures as well as other aspects of epilepsy. Many reviews have outlined the evidence of the beneficial effect of PA on mood, QOL, seizures, and cognition in PWE but few have explored the effect of PA specific to older adults with epilepsy. The older adult population has unique physiology and comorbidities, as well as the potential for increased benefit from PA that necessitates examining this unique population in greater detail.

The aim of the following narrative review is to summarize the available literature on PA in older adults with epilepsy. We will examine the potential benefits of PA for epilepsy-associated comorbidities and seizures, including both direct antiseizure effects, and the role of PA in reducing the impact of causative factors for seizures in older adults. We will present potential mechanisms for these actions, highlight areas in need of future investigation, and provide recommendations on the promotion on PA in this population.

## 2 Current landscape

In recent years, we have observed an increase in publications investigating PA in the epilepsy population [7]. Many studies have shown that PWE are less physically active than people without epilepsy [8], [9], [10]. However, the PA levels of older adults with epilepsy compared to older adults without epilepsy have not been specifically evaluated. From studies in the general population, we know that many older adults do not meet the Physical Activity Guidelines for Americans (PAG), with one study showing only 59.7 % of American adults aged 60–69 years old meeting the guidelines as measured by self-report, and only 8.5 % as measured by accelerometry data, with even lower percentages for those 70 years old or older [11]. Indeed, the PAG are challenging for many populations to meet, but studies have shown increasing sedentary time and a decline in moderate to vigorous activity as people age [12], making this a particular concern for older adults with epilepsy.

PA has the potential to improve many conditions that are highly prevalent in the epilepsy population, such as mood disorders and cognitive impairment. Small studies have shown improvement in various measures of mood and emotional well-being in PWE following a PA intervention [13], [14], [15], [16]. PA is also one of the few interventions that has demonstrated improvement in formal cognitive function assessment in small samples of adults with epilepsy, showing improvement in verbal learning and memory, global cognitive function, executive attention, and verbal fluency [14], [17]. However, these studies did not specifically assess outcomes in older adults with epilepsy, who are more likely to have other contributing factors to cognitive dysfunction such as comorbid neurodegenerative disorders [18], cerebrovascular disease [19], and/or polypharmacy [20].

In 2022, the first meta-analysis of the effects of PA on seizure frequency and QOL in adults with epilepsy was performed. There was a significant improvement in QOL following a PA intervention in the pooled analysis. The authors found a non-significant decrease in seizure frequency in the pooled analysis, which was limited by heterogeneity and small sample size [6]. This did not include data from the more recently published randomized controlled study by Kaur et al, which demonstrated significantly higher odds of > 50 % seizure reduction (OR 4.1) and complete seizure remission (OR 7.4) with a yoga intervention compared to control [16]. Notably, the mean (SD) age in this study was 29.64 (8.34), so the applicability to older adults is not yet determined.

Despite increasing research in PA in PWE, none have specifically examined an older adult population. Most studies report an age range, mean age, and standard deviation, but no information is provided that allows for analysis of outcomes specific to older adults, or even information on how many older adults were included. In a recent clinic-wide survey of PA behaviors in 95 adults with epilepsy [21], only ten respondents (10.5 %) were over the age of 65. However, adults over 65 years old make up over 17 % of the United States population. This highlights an important issue: unless a directed effort is made to include the older adult population in these studies, representation of this age group is likely to be lacking, thereby limiting the generalization of results to this cohort despite their great potential to benefit from PA.

## 3 Physical activity and vascular health in epilepsy

The increased prevalence of cardiovascular disease and sudden cardiac arrest in PWE [22], [23] has led to the concept of the “epileptic heart.” This condition is characterized by features such as interstitial fibrosis, altered autonomic tone, hyperlipidemia, accelerated atherosclerosis, and increased risk of cardiac arrhythmia. These changes are thought to be due to repeated catecholamine surges and hypoxemia from seizures, adverse effects of ASMs, and genetic factors [24]. PWE face a heightened risk of premature death, with cardiac comorbidities contributing to this risk beyond sudden unexpected death in epilepsy (SUDEP) [25]. Additionally, several studies have shown that PWE have an increased risk of stroke. In a systemic review of five large population cohort studies from across four different countries, PWE demonstrated an increased risk of stroke with hazard ratios ranging from 1.1 to 2.85 [26], with the highest risk seen in the study that included only older adults with late onset seizures (>60 years old) [27].

It is well documented in the general population that PA can reduce the risk of cardiovascular disease, stroke, heart failure, and cardiovascular disease-related mortality, in a dose-dependent manner [28], [29]. In fact, at least for cardiovascular disease, the greatest benefit is seen early in the dose–response relationship, suggesting that any increase in PA has a significant impact on risk reduction [30], [31]. This is a key finding when encouraging PA in older adults, who may be less able to perform the amount or intensity of PA suggested by the guidelines.

In terms of epilepsy prevention, increasing PA has been shown to decrease the risk of stroke [31], and therefore may lessen the incidence of post-stroke epilepsy. PA also has indirect benefits through the modulation of several epilepsy risk factors like cerebrovascular disease and Alzheimer’s disease [32]. One large scale cohort study found that cerebrovascular disease accounted for 15 % of newly diagnosed epilepsy across all age groups and nearly 50 % were older adults [33]. Similar studies reported incidence of stroke-related seizures ranging from 2 % to 20 % in all ages [34]. Several risk factors related to the development of epilepsy after stroke are age (<65 years), hyponatremia, hemorrhagic subtype of stroke, alcohol use, cortical involvement, stroke severity and early seizures (within the first week) after stroke [34]. A multinational case–control study has further found physical inactivity to be the second most important risk factor for stroke [35]. One review showed that hyperglycemia, type 1 or 2 diabetes, dyslipidemia, hypertension, cardiovascular morbidities, peripheral infections, early seizures, major depression, use of antidepressants, use of statins, and pre-existing dementia might contribute to the development of epilepsy after ischemic stroke [36].

Finally, PA interventions in adults with epilepsy have shown improvement in lipid profiles, body fat [37], autonomic tone [38], and cardiorespiratory fitness [39], [40]. Given the increasing risk for cardiovascular disease with increasing age, PA may be even more important in older adults with epilepsy, though the effects specific to older adults have not been specifically studied.

## 4 Physical activity and neuroinflammation in epilepsy

Neuroinflammation is defined as the activation of immune cells and inflammatory mediators within the central nervous system in response to various insults such as trauma, tissue damage, infection, autoimmune conditions, seizures, and stress. In epilepsy, an increased inflammatory response is observed in both serum and in epileptogenic tissue [41], and has been implicated both in epileptogenesis and the progression of epilepsy [42], [43]. Several key inflammatory mediators of the immune system including interleukin-1β (IL-1β), transforming growth factor-β (TGF-β), high-mobility group box protein 1 (HMGB1), leukotrienes, and Toll-like receptor (TLR) may play a role in epileptogenesis [44], [45]. Additionally, it is proposed that neuroinflammation might contribute to the development and progression of comorbidities like anxiety and depression in epilepsy [46].

Several anti-inflammatory agents are used in the treatment of epilepsy to reduce seizures [42], [47]. Anti-inflammatory medications like adrenocorticotropic hormone, glucocorticoids, vigabatrin (an anti-seizure medication with anti-inflammatory properties), and approaches like the ketogenic diet (high fat and low carbohydrate/ low protein) have been shown to reduce seizure frequency [43]. While the mechanism of the ketogenic diet in reducing seizures is not fully understood, byproducts of fatty acid metabolism, including ketone bodies, are generally thought to reduce neuroexcitation and neuroinflammation [48], [49]. Additionally, several preclinical studies have shown that leukotriene receptor antagonists can reduce the incidence and severity of seizures [50]. In humans, a retrospective cohort study showed a reduced rate of seizure-related hospitalizations in those starting leukotriene receptor antagonists for asthma or allergies in 60–89 year olds, suggesting that targeting this type of inflammation in older adults may improve seizures [51]. With the slow deterioration of homeostatic functions that occur with aging, the balance between pro-inflammatory and anti-inflammatory cytokines tends to shift towards a pro-inflammatory state. As such, it makes sense that the role of neuroinflammation may be of particular importance in older adults with epilepsy.

PA positively effects homeostasis in the body, including in the neural tissue, by modulating gene expression and increasing production of mediators involved in energy metabolism, anti-oxidation, and anti-inflammation [52]. The beneficial effects of PA have been demonstrated in neurodegenerative diseases such as multiple sclerosis, Alzheimer’s disease, and Parkinson’s disease, where the contribution of acute and chronic inflammation is well recognized. In epilepsy, several preclinical and clinical studies show that PA can decrease seizure burden [8], [53], [54], and one possible mechanism of this effect is through the mediation of neuroinflammation [53].

Animal studies have shown that endurance training in mice prior to seizure induction delayed the onset of seizures, decreased inflammatory markers in the hippocampus (IL-1β, TNF-α) and ameliorated the effects of oxidative stress [55]. Furthermore, trained epileptic mice taught to swim showed increased major antioxidants (superoxide dismutase and catalase) and Na^+^, K^+^-ATPase activity in the brain along with a reduction in seizure duration [56]. In addition, PA can reduce seizure severity and levels of pro-inflammatory cytokines (IL-1β, TNF-α) in the cortex, but not in the hippocampus in mouse models of epilepsy [57].

Additional mechanisms proposed to explain the effects of PA include the regulation of oxidative stress, reduction in apoptosis, modulation of the blood–brain barrier (BBB), and regulation of glutamatergic activity [58]. PA is well known to upregulate antioxidant levels and mitigate the effects of reactive oxygen species (ROS) that are produced during seizures [59]. In animals studies, exercise exposure was associated with reduced seizure susceptibility, higher antiapoptotic signaling, and decreased proapoptotic response [60]. Due to blood brain barrier (BBB) breakdown, single seizures or status epilepticus can induce neuronal injury, which may be assessed using various blood and cerebrospinal fluid biomarkers [61], [62]. Research has demonstrated that PA reduces BBB permeability through direct mechanisms, such as improved endothelial function, and indirect mechanisms, such as reduced systemic inflammation [62], [63]. In animal models, PA has been shown to ameliorate glutamatergic activity in rats following kainite-induced seizures [64].

In humans, studies investigating the physiological effects of PA in the prevention and treatment of epilepsy are limited. A retrospective cohort study in humans found that PA reduces incidence of focal, but not generalized epilepsy [65]. The authors then examined this effect in a genetically modified mouse model and found that PA prevented or at least delayed the development of epilepsy but did not affect seizure frequency after seizures developed. PA increased hippocampal neurogenesis in rat brains (without changes in BDNF or TrkB expression) but did not change astrocytic and microglial activation. Importantly, the role of BDNF/TrkB cascade has yet to be clearly explained given contradictory findings in animal studies of epileptogenesis and ictogenesis [52], [66], [67]. Further animal and human studies are needed to understand the mechanisms by which PA may directly benefit seizures and how that applies to the older adult population.

## 5 Physical activity and cognition in epilepsy

There is strong evidence that PA positively impacts cognition in healthy older adults [68]. This effect is multifactorial, as numerous studies have shown that PA is associated with reduced inflammation, improvements in mood and anxiety, increased cerebral blood flow, and higher concentrations of beneficial neurotransmitters. While the specific factors driving improvements in particular cognitive domains are not fully understood, it is likely a combination of these mechanisms that contributes to the observed cognitive benefits. Notably, PA appears to be especially beneficial for individuals with existing cerebral dysfunction, as significant cognitive improvements are seen in this population. In two PA studies using cognitive testing and fMRI outcomes comparing older adults with MCI and age matched healthy participants, improvements were seen in cognitive performance and functional brain connectivity in the MCI groups following a supervised PA program [69], [70].

In epilepsy, cognitive impairment is common, has a significant impact on QOL, and has limited treatment options. In a systematic review of six studies evaluating the association of PA and cognition in PWE, all showed a positive association between PA and cognition and 2 longitudinal studies of PA interventions showed improvement in at least one cognitive domain [71]. Specific effects in older adults with epilepsy, however, were not evaluated.

## 6 Physical activity and fall prevention

Falls are the most prevalent injury among adults aged 65 years and older, representing a significant health concern [72]. In any given year, older adults face about a 27 % likelihood of experiencing a fall [73] and falls are the most common cause of traumatic brain injury (TBI) within the older adult population [74]. A serious sequela of TBI is post-traumatic epilepsy, accounting for 10–20 % of epilepsy cases in the general population [75]. Therefore, reducing the incidence of falls and head trauma may help to prevent acquired epilepsy in older patients. Another study showed that PA can decrease the fall rate by 23 % in older individuals, thereby reducing associated complications [76].

In evaluating various types of PA, several studies have evaluated the effects of PA modalities on fall risk, balance, and physical performance. One multinational project, the Otago Exercise Program, conducted in the US, Turkey, and Greece, was found to be effective in improving balance and reducing falls among older adults in long-term care facilities [77], [78], [79], [80]. Another study evaluated an in-home, 8-week PA program in older adults and showed improved balance, increased muscle strength and reduced fall risk [81]. In a systematic review of PA interventions for falls prevention in community-dwelling older adults [82], interventions that were most effective in decreasing the frequency of falls included balance and functional training interventions, Tai Chi, and programs with multiple PA modalities (multicomponent interventions). Considering these insights, promoting PA tailored to the needs of the older adults may aid in the prevention of falls and head trauma that can contribute to the development of epilepsy. Whether PA can help improve fall risk specifically in older adults with epilepsy has not been studied.

## 7 Physical activity and mental health in epilepsy

There is exponential growth in the diagnosis of mental health disorders including depression and anxiety. According to a report from 2019, one in eight people, or 970 million cases of mental disorders were recognized globally, with depressive and anxiety disorders being the primary contributors to this burden [83], [84], [85]. The COVID-19 pandemic has increased psychological distress to the range of 35 %-38 % and moved mental health up the list of global health priorities [85], [86]. Importantly, PWE exhibit a much higher rate of mood disorders than the general population [87], which may be even higher in older adults with epilepsy [88].

PA drives the release of β-endorphins and steroids into the bloodstream which has a positive impact on mood [89], [90]. Regular PA can also improve self-esteem and psychological well-being [40]. In a study conducted on thirty-two physically active, cognitively normal older adults in which participants were engaged in a 30-minute moderate intensity cycling exercise, the authors demonstrated that aerobic exercise enhances mood by altering the functional-connectivity between the anterior insula, a key area in the cingulo-opercular network, and the hippocampus [91]. The cingulo-opercular network is one of the largest human brain networks, with inputs directly involving motor control [92] and pain [93]. Dysfunction of this network is associated with mood disturbance including depression [94] and anhedonia [95]. Incorporating PA into day-to-day life has repeatedly been shown to improve mental health [96] and has been proposed as an adjunct treatment for depression and anxiety [97], [98], [99], [100], [101], [102].

Several studies have explored how PA effects mood and psychological well-being in PWE. One of the first randomized controlled studies of exercise in PWE included 23 patients (n = 14 in the exercise group) and found improved mood in the exercise group after a 12-week exercise intervention [13]. A more recent randomized controlled trial of a 12-week combined exercise intervention in 21 participants found improved stress in the exercise group but no significant change in depression or anxiety scores [103]. A recent larger trial of 78 participants undergoing a 12-week walking physical activity intervention reported improved depression and anxiety scores in the intervention group compared to the control group [104]. None of these studies, however, included participants 65 years or older.

## 8 Physical activity and adverse effects of anti-seizure medications

Adverse effects of ASMs are common, occurring in up to 88 % of people taking ASM, and include dizziness, imbalance, cognitive dysfunction, worsened mood, sleepiness, fatigue, and low Vitamin D and impaired bone mineral density [105], [106], [107]. Even with the development of newer, second-generation ASMs, overall treatment tolerability remains low [108]. PA has the potential to improve many of these adverse effects such as cognition, mood, balance, and falls for the reasons discussed above. Whether PA can specifically improve ASM side effects in PWE is still being studied, but one small study showed that a 12-week PA intervention improved scores on the Adverse Event Profile (AEP), a measure of anti-seizure medication adverse effects [103].

It is well-established that PA, especially weight-bearing PA, improves bone health and prevents against osteopenia and osteoporosis. The mechanism of this effect is complex, but has been noted in children, adults, and especially in women [109]. Despite this known benefit and the increased risk of fractures in PWE over the age of 50, they are still less likely to engage in PA than other older adults [110]. Since adverse effects on bone health can be seen in as short a time as 1–2 years of use with some ASMs [111], preventive measures may be useful even for older adults with new-onset epilepsy.

Dizziness and imbalance are common adverse effects of ASMs and are usually dose-related [112]. Although vestibulocerebellar adverse effects are more common with sodium-channel acting drugs, most ASMs can cause them. Reducing the dose can help manage these adverse effects but may not always be an option to maintain acceptable seizure control. Furthermore, some ASMs may cause permanent dysfunction of balance control mechanisms. In one study, there was no immediate improvement in balance with discontinuation of drugs in a hospital seizure-monitoring unit over an interval of a few days [113]. In another study, chronic users of ASMs appeared to have progressive loss of balance function in comparison to twin and sibling controls [114]. In older adults with epilepsy, ASM use contributes to an increased risk of falls, which can result in serious injury or death [115]. As discussed above, PA can reduce the risk of falls in older adults and should be considered to help prevent injury and fall related co-morbidities in older adults with epilepsy.

## 9 Conclusion and future directions

There is a lack of evidence specifically evaluating PA in older adults with epilepsy. Existing studies examining PA in PWE are heterogenous and vary not only in the modality, intensity, and duration of the PA intervention, but also the outcome measures. In many PA studies in PWE, older adults are excluded. When older adults are included, the specific number or proportion of older adults is often not reported, and/or age is not included as a covariate, limiting the generalizability to older adults with epilepsy. Despite the current lack of direct evidence supporting PA for older adults with epilepsy, there are many significant expected health and QOL benefits. These include seizure control, seizure prevention, cardiovascular and cerebrovascular health, mood, cognition, and falls prevention. Therefore, we advocate for the development of tailored PA programs that accommodate the unique needs of older adults with epilepsy. Older adults with limited mobility have unique challenges in carrying out PA regimens which are often designed for a younger audience. While it is well documented that participation in physical activity programs is safe for older adults, [4] it is important to consider the specific physical function and medical comorbidities of each individual participant when designing a physical activity intervention for any age group. Older adults are statistically more likely to have comorbid cardiovascular, respiratory, or musculoskeletal comorbidities which may therefore impact that type of physical activity that is safest and most effective for their needs [116]. Multicomponent physical activity programs which include aerobic, muscle strengthening, balance and/or gait and physical function training are often most effective in meeting the needs of older adults [4]. PA program adaptations for older adults may include group programs to create a safe and welcoming environment and promote participation. Methods for transport to and from PA programs should be funded where possible due to possible driving limitations related to seizures and/or age-related comorbidities. Finally, self-led PA is increasingly accessible and may be conducted by video-link with online guidance which may require additional instruction and technical support for this population.

Interdisciplinary collaborations are important to create comprehensive care plans that include PA as a key component for managing epilepsy in older adults. This includes education sessions among staff and clinicians, the distribution of literature to patients on the benefit of PA, and recommendations of useful resources. A key component in the advancement of PA programs in older adults with epilepsy is to continually educate all stakeholders that PA is generally safe in PWE, including in older adults, and should be encouraged at every opportunity. Clinicians have a responsibility to promote PA among their patients and particularly in epilepsy medicine, and we believe increased PA has a high potential to improve outcomes in older adults with epilepsy.

## Ethical statement

We confirm that the following review article adhered to all relevant ethical guidelines. None of the authors have any conflicts of interest. All authors contributed to the preparation of this manuscript.

## CRediT authorship contribution statement

**Stephen P. Klaus:** Writing – review & editing, Writing – original draft, Supervision. **Serdar Akkol:** Writing – original draft, Writing – review & editing. **Smitha K. Achuthan:** Writing – original draft. **Annie He:** Writing – original draft. **Cynthia Zheng:** Writing – original draft. **Ed Faught:** Writing – review & editing, Writing – original draft. **Halley B. Alexander:** Writing – review & editing, Writing – original draft.

## Funding

This work was partially supported by the Department of Veterans Affairs VISN 6 Career Development Award.

## Declaration of competing interest

The authors declare that they have no known competing financial interests or personal relationships that could have appeared to influence the work reported in this paper.
